# When Formulas Fail: A Secondary Analysis of Dual-Energy X-ray Absorptiometry (DXA) Body Composition Data Comparing Ideal and Lean Body Weight Equations Against Measured Lean Body Weight in Obese Children

**DOI:** 10.7759/cureus.96759

**Published:** 2025-11-13

**Authors:** Mike Wells, Lara N Goldstein

**Affiliations:** 1 Department of Emergency Medicine, Florida Atlantic University Charles E. Schmidt College of Medicine, Boca Raton, USA; 2 Department of Emergency Medicine, Memorial Healthcare System, Hollywood, USA

**Keywords:** foster formula, ideal body weight, lean body weight, mclaren formula, moore formula, pediatric weight estimation, peters formula

## Abstract

Background

Accurate weight-based drug dosing in obese children is essential for safe and effective therapy, yet the relationships between ideal body weight (IBW), lean body weight (LBW), and actual body composition remain poorly defined. IBW was originally developed to assess nutritional status rather than guide drug dosing, but it is often used as a surrogate for LBW despite limited validation. This study aimed to compare commonly used IBW and LBW formulas against measured LBW to evaluate their accuracy and clinical applicability in obese children.

Methods

A secondary analysis was conducted using data from 30 obese children (BMI-for-age Z-score ≥2.0) obtained in a hospital-based pediatric body composition study. Anthropometric data and dual-energy X-ray absorptiometry (DXA) measurements of LBW were available. Seven published equations for IBW and LBW estimation were evaluated. Accuracy was assessed using mean percentage error, root mean square percentage error, and proportions of estimates within 10% (P10) and 20% (P20) of measured LBW.

Results

All IBW formulas substantially overestimated measured LBW, with mean biases ranging from 18% to 30%, and none achieved acceptable accuracy (P10 <70%). Among LBW equations, the Foster formula demonstrated the highest accuracy, while the Peters and Callaghan methods performed poorly. Adjusting IBW formulas with simple correction factors improved LBW prediction accuracy, though similar corrections were not feasible for the Peters and Callaghan methods.

Conclusions

IBW formulas cannot reliably substitute for LBW in obese children without modification. The Foster equation offers the best accuracy for LBW estimation, while the BMI₅₀ IBW method shows potential for adaptation in clinical tools. Further validation of hybrid or adjusted models is required to optimize pediatric drug dosing safety.

## Introduction

Background

In 2022, more than 390 million children and adolescents aged 5 to 19 years were classified as overweight globally, including approximately 160 million with obesity [1]. In the United States, approximately 20% of children and adolescents have obesity [2]. Concerningly, children with higher BMIs are significantly more likely to utilize the ED than children with a healthy weight, independent of confounding factors. They also have a greater number of ED visits related to accidents, injuries, and acute respiratory illnesses compared with underweight children or children with a healthy weight [3]. Consequently, clinicians in hospital and emergency settings frequently need to calculate weight-based medication doses for children with obesity. This process requires an understanding of the intricacies and pitfalls of the dose calculation mechanics to avoid potentially harmful dosing errors [4-6].

In children with obesity, drug doses should be calculated differently from those in children with normal weight. According to the World Health Organization, lipophilic drugs should be scaled according to total body weight (TBW), and hydrophilic drugs should be scaled to ideal body weight (IBW) [7]. This strategy is intended to ensure that therapeutic doses of lipophilic drugs are administered and that overdoses of hydrophilic drugs are avoided. However, many experts suggest that lean body weight (LBW), and not IBW, is the best weight scalar for hydrophilic drugs [8]. In practice, IBW is often preferred over LBW as a dose scalar, as it is easier to calculate, but, in reality, both IBW and LBW are challenging to calculate in children [9,10]. However, the agreement between different methods of calculating IBW has been shown to be poor, especially in older children and at the extremes of length and habitus [11,12]. Furthermore, the biological validity of IBW as a dose scalar has not been proven. Although LBW and IBW are often used interchangeably for dosing in obese children, the relationship between IBW and LBW in children has not been investigated adequately [13]. In fact, IBW calculation methods were never validated for drug dosing but simply repurposed from other indications, such as nutritional assessment.

Importance

Incorrect dosing in obese children may directly result in worse clinical outcomes [10,14,15]. At least part of this is from incorrect dose scaling. It is evident that arbitrary, unscientific dose reductions in obese children are not appropriate, but it is also evident that the use of IBW might not be ideal for this purpose. An understanding of the relationship between IBW and LBW would provide insight into the validity of IBW as a dosing scalar. In addition, LBW is frequently calculated from IBW. This is problematic if the assumed relationship between LBW and IBW is not as expected [16]. Ultimately, an evidence-based understanding of IBW and its relationship to body composition in obese children is a critical matter of patient safety.

Goals of this study

This study aimed to evaluate the accuracy of commonly used IBW and LBW estimation formulas in obese children by comparing them to dual-energy X-ray absorptiometry (DXA)-measured LBW, using a secondary analysis of existing pediatric body composition data, to determine which methods are most appropriate for clinical drug dosing applications.

## Materials and methods

Study design and setting

This was a secondary analysis of subgroup data from a previous pediatric and adolescent weight estimation study conducted in the ED of Netcare Clinton Hospital, an academic pediatric hospital in Johannesburg, South Africa, between October 2014 and January 2015 [17]. This sample population was chosen to represent a clinically relevant obesity phenotype in a relevant prescribing context.

Participants

The original study recruited a convenience sample of 332 children ≤18 years of age. For the secondary analysis, the data for 30 obese children (BMI-for-age Z-score ≥2.0) were extracted from the sample. A sample size analysis for two-tailed, paired analysis of proportional data showed that a sample of 29 participants would be required to detect a 10% absolute difference in accuracy, with a power of 0.8 and statistical significance of 0.5. The available sample was therefore of sufficient size to show clinically meaningful differences.

**Figure 1:**
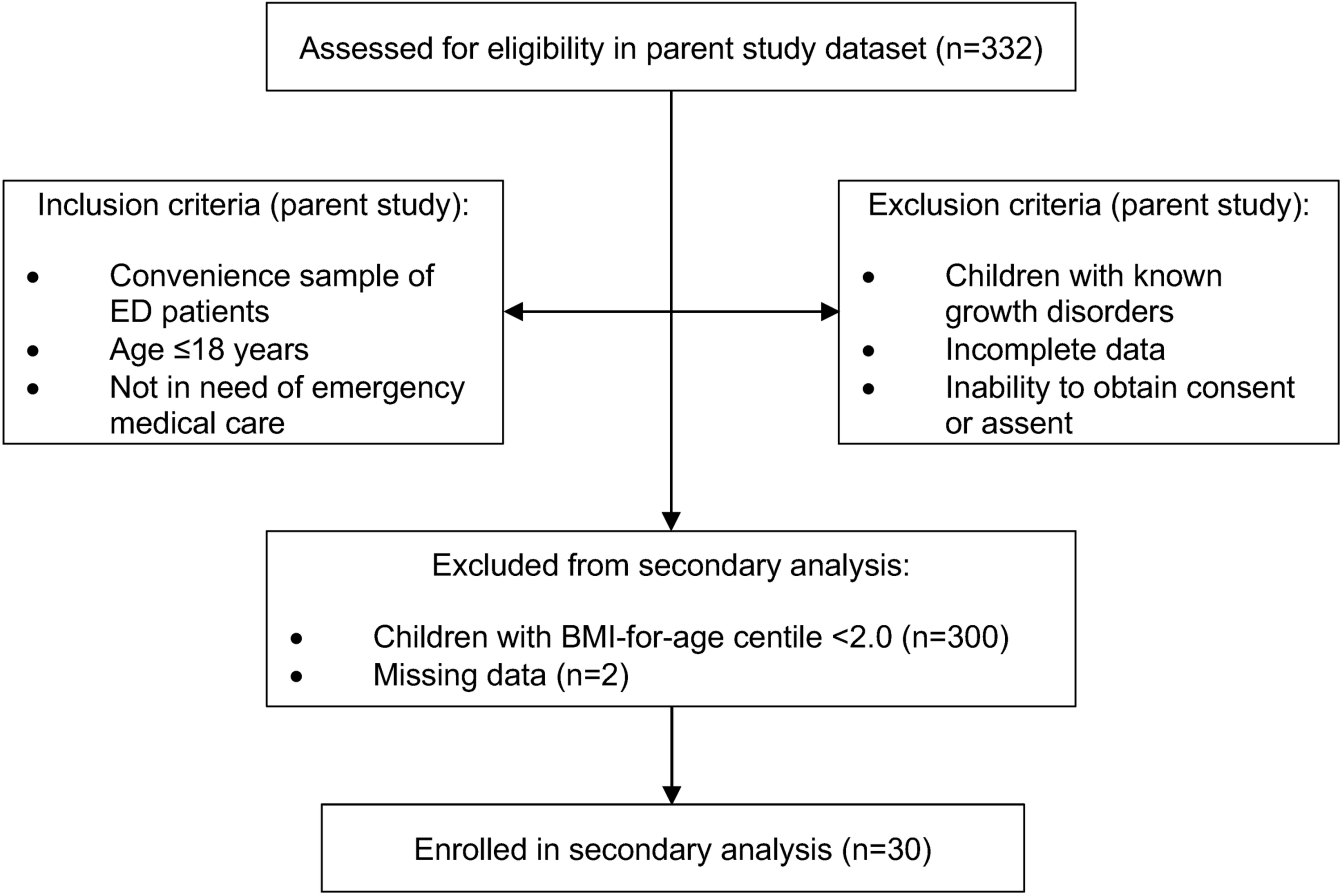
Cohort flowchart for this study The inclusion and exclusion criteria for the parent study are shown, as well as the exclusion criteria used to obtain the sample for this secondary analysis.

The cohort flowchart illustrating the inclusion and exclusion criteria for patients in the present study is shown in Figure 1.

Data collection

In the parent study, anthropometric measurements (recumbent length, mid-arm circumference, and measured weight) and DXA measurements of body composition were obtained for each participant. A Hologic Discovery A densitometer, with pediatric software provided by the manufacturer, was used to acquire the DXA measurements.

Data analysis

IBW and LBW values were calculated for each child, using seven common existing methods (Table 1) [12,18]. These included the Foster, Peters, and Callaghan formulas for estimating LBW and the BMI₅₀, Moore, McLaren, and Traub-Johnson formulas for estimating IBW. The equations were uniformly applied as originally described in the literature, as appropriate for the sex and age of the participant.

**Table 1:** Methods for estimating IBW and LBW evaluated in this study DXA, dual-energy X-ray absorptiometry; IBW, ideal body weight; LBW, lean body weight; TBW, total body weight

Method	Description
BMI₅₀method (IBW in kg)	Plot the child’s BMI on a BMI-for-age growth chart. Read off the BMI at the 50th centile at that age. Multiply that BMI by the square of the child’s height (in meters).
McLaren method (IBW in kg)	Plot the child’s height on a height-for-age growth chart. Draw a horizontal line to the 50th centile. Identify the age at that point on the 50th centile. Look up the 50th centile weight at that age on a weight-for-age growth chart.
Moore method (IBW in kg)	Plot the child’s height on a height-for-age growth chart and note the centile. Read the weight at the same centile and age on a weight-for-age chart.
Traub-Johnson formula (IBW in kg)	$IBW=2.05\times\ e^{(0.02\ \times\ length)}$
	Length is measured in cm. This formula was specifically developed to predict weight at the 50th centile of weight-for-height, essentially the “standard child” weight.
Peters formula (LBW in kg)	$LBW=0.0817\times\ {TBW}^{0.6469}\ \times\ {height}^{0.7236}$
	TBW is total body weight measured in kg and height in cm
Foster formula (LBW in kg)	Female: $\ln{\left(LBW\right)}=-3.8345+\ 0.954\times\ln{\left(height\right)\ +0.6515\times\ln{\left(TBW\right)}}-0.0102\times{BMI_z}^2​​​​​​$​
	Male: $\ln{\left(LBW\right)}=-2.8990+\ 0.8064\times\ln{\left(height\right)}+0.5674\times\ln{\left(TBW\right)}+0.0000185\times{TBW}^2-0.0153\times{BMI_z}^2+0.0132\times age$
	Height is measured in cm, TBW in kg, age in years, and BMI_z_ is the BMI-for-age Z-score. The equation for females has two fewer variables than the one for males. The natural logarithm (ln) is used in the equations.
Callaghan method (LBW in kg)	$LBW=IBW+\ 0.29\ \times\ (TBW-IBW)$
	This method uses the BMI₅₀ for the IBW estimate. All weights are in kg.
DXA body composition analysis (LBW in kg)	Measurement of LBW: one of the gold standard reference methods of body composition analysis. The Hologic Discovery A densitometer was used with the manufacturer’s software for pediatric body composition analysis. In this study, we used the term LBW to refer to lean mass, or fat-free mass. The terms fat-free mass and LBW are generally regarded as synonymous.

These estimated values were then compared against the gold standard DXA-measured LBW to quantify the association between the estimates and actual LBW. Standard statistical methods for weight estimation studies (method comparison studies) were used to describe the associations. The mean percentage error (MPE) was used as a measure of bias, the root mean square percentage error (RMSPE) was used as a measure of precision, and the percentages of estimates within 10% and 20% of the measured LBW (P10 and P20, respectively) were used as measures of overall accuracy. An acceptable accuracy outcome was defined to be a P10>70% and a P20>95% [19]. Subgroup analyses were performed to detect any differences in the accuracy of predictions between males and females.

The analyses were performed using Excel for Mac (Microsoft Office 365 Version 16.73, Released 2023; Microsoft Corporation, Redmond, WA, USA) and IBM SPSS Statistics for Windows, Version 31.0 (Released 2025; IBM Corp., Armonk, NY, USA). Comparisons of P10 and P20 proportional data were made using the chi-square or Fisher’s exact test (as appropriate for expected cell sizes), and odds ratios with 95% confidence intervals were calculated. Comparisons between RMSPE continuous data were made using Student’s t-test (two-tailed, independent samples) for normally distributed data. Tests for normality were conducted using the Kolmogorov-Smirnov test. A significance level of p < 0.05 was used for all analyses.

Ethics

This research was approved by the Human Research Ethics Committee of the University of the Witwatersrand, Johannesburg, South Africa. Assent was obtained from children, and written informed consent was obtained from parents or guardians.

## Results

The 30 children included 14 males (46.7%), had a median age of 6.8 years (range: 1.4-15.0 years, with 20 children <8 years of age (66.7%)), a median length of 124.5 cm (range: 82.0-176.5 cm), a median TBW of 30.8 kg (range: 14.4-94.0 kg), a median BMI-for-age Z-score of 2.3 (range: 2.0-4.2), and a median measured LBW of 21.1 kg (range: 7.9-58.5 kg).

The predictive performance of each LBW formula for estimating LBW is shown in Table 2. The MPE data was not compared statistically, as this reflects only the bias of the method and not the overall performance. The P10 and P20 data are proportional and were statistically evaluated using the chi-square test (or the Fisher’s exact test). The RMSPE data were normally distributed (as determined by the Kolmogorov-Smirnov test), and statistical comparisons were therefore made using the paired t-test. The Foster method predicted LBW with good accuracy. The Peters and Callaghan methods were not accurate, overestimating LBW by 27.5% and 33.1%, respectively, which could increase hydrophilic drug doses by clinically meaningful margins. The Foster formula performed equally well in males and females (there were no significant differences in PW10 and RMSPE). However, both the Peters and Callaghan methods were significantly less precise in females than in males, overestimating LBW by 7.9% and 7.3% more in females for each method, respectively, making females at higher risk for significant dosing errors than males.

**Table 2:** Results of the comparisons between the different calculations for estimating LBW and measured LBW A positive value of MPE indicates an overestimation of LBW. The results of Fisher’s exact tests between the P10s of the male and female participants are indicated by superscripts: ^a^ OR 1.5 (0.3, 7.9) p = 0.613, ^b^ NaN p = 1, ^c^ NaN p = 1. The results of the t-tests between the RMSPE of the male and female participants are indicated by superscripts: ^d^ t-test (two-tailed, independent samples) p = 0.513, ^e^ t-test (two-tailed, independent samples) p = 0.02, ^f ^t-test (two-tailed, independent samples) p = 0.047. LBW, lean body weight; LLOA, lower 95% limit of agreement; MPE, mean percentage error; NaN, not a number (cell size too small for analysis); PW10, percentage of estimates within 10% of measured fat-free mass; PW20, percentage of estimates within 20% of measured fat-free mass; ULOA, upper 95% limit of agreement

Parameter	Foster formula			Peters formula			Callaghan formula		
	All	Male	Female	All	Male	Female	All	Male	Female
MPE	-2.8	-3.3	-2.3	27.5	23	31.3	33.1	29.5	36.2
LLOA	-22.4	-21.3	-23.9	7.7	7.1	11.3	14.3	15.2	15.9
ULOA	16.9	14.7	19.2	47.2	38.9	51.4	51.8	43.7	56.6
PW10	73.3	78.6^a^	68.8^a^	0	0^b^	0^b^	0	0^c^	0^c^
PW20	96.7	100	93.8	33.3	42.9	25	0	0	0
RMSPE	10.3	9.5^d^	10.9^d^	29.2	24.3^e^	32.9^e^	34.4	30.3^f^	37.6^f^

The performance of each IBW method for estimating LBW is shown in Table 3. None of the methods showed a close association. All IBW formulas had a reasonably consistent overestimation bias of 15-25%. There were relatively minor differences in the performance of the various methods between males and females.

**Table 3:** Results of the comparisons between the different calculations for estimating IBW and measured LBW A positive value of MPE indicates an overestimation of LBW. The results of Fisher’s exact tests between the P10s of the male and female participants are indicated by superscripts: ^g^ OR 1.2 (0.2, 7.1) p = 0.855, ^h^ OR 0.5 (0.04, 6.7) p = 0.626, ^i^ NaN p = 1, ^j^ OR 1.2 (0.1, 20) p = 0.922. The results of the t-tests between the RMSPE of the male and female participants are indicated by superscripts: ^k^ t-test (two-tailed, independent samples) p = 0.174, ^l^ t-test (two-tailed, independent samples) p = 0.042, ^m^ t-test (two-tailed, independent samples) p = 0.977, ^n^ t-test (two-tailed, independent samples) p = 0.111. IBW, ideal body weight; LBW, lean body weight; LLOA, lower 95% limit of agreement; MPE, mean percentage error; NaN, not a number (cell size too small for analysis); PW10, percentage of estimates within 10% of measured fat-free mass; PW20, percentage of estimates within 20% of measured fat-free mass; ULOA, upper 95% limit of agreement

Parameter	BMI₅₀method			McLaren method			Moore method			Traub-Johnson formula		
	All	Male	Female	All	Male	Female	All	Male	Female	All	Male	Female
MPE	18.7	16.5	20.7	22.3	18.1	26	30	30.7	29.4	21	19.1	22.7
LLOA	-0.8	0.3	-1.1	0	5.5	-0.6	8.5	12.7	4.7	4.3	9.2	1.9
ULOA	38.2	32.6	42.4	44.6	30.7	52.6	51.6	48.8	54.2	37.8	28.9	43.6
PW10	20	21.4^g^	18.8^g^	10	7.1^h^	12.5^h^	3.3	0^i^	6.3^i^	6.7	7.1^j^	6.3^j^
PW20	56.7	50	62.5	43.3	57.1	31.3	20	21.4	18.8	60	71.4	50
RMSPE	21.1	18.3^k^	23.3^k^	24.9	19.1^l^	29.1^l^	31.9	32.0^m^	31.9^m^	22.7	19.7^n^	25.0^n^

**Figure 2:**
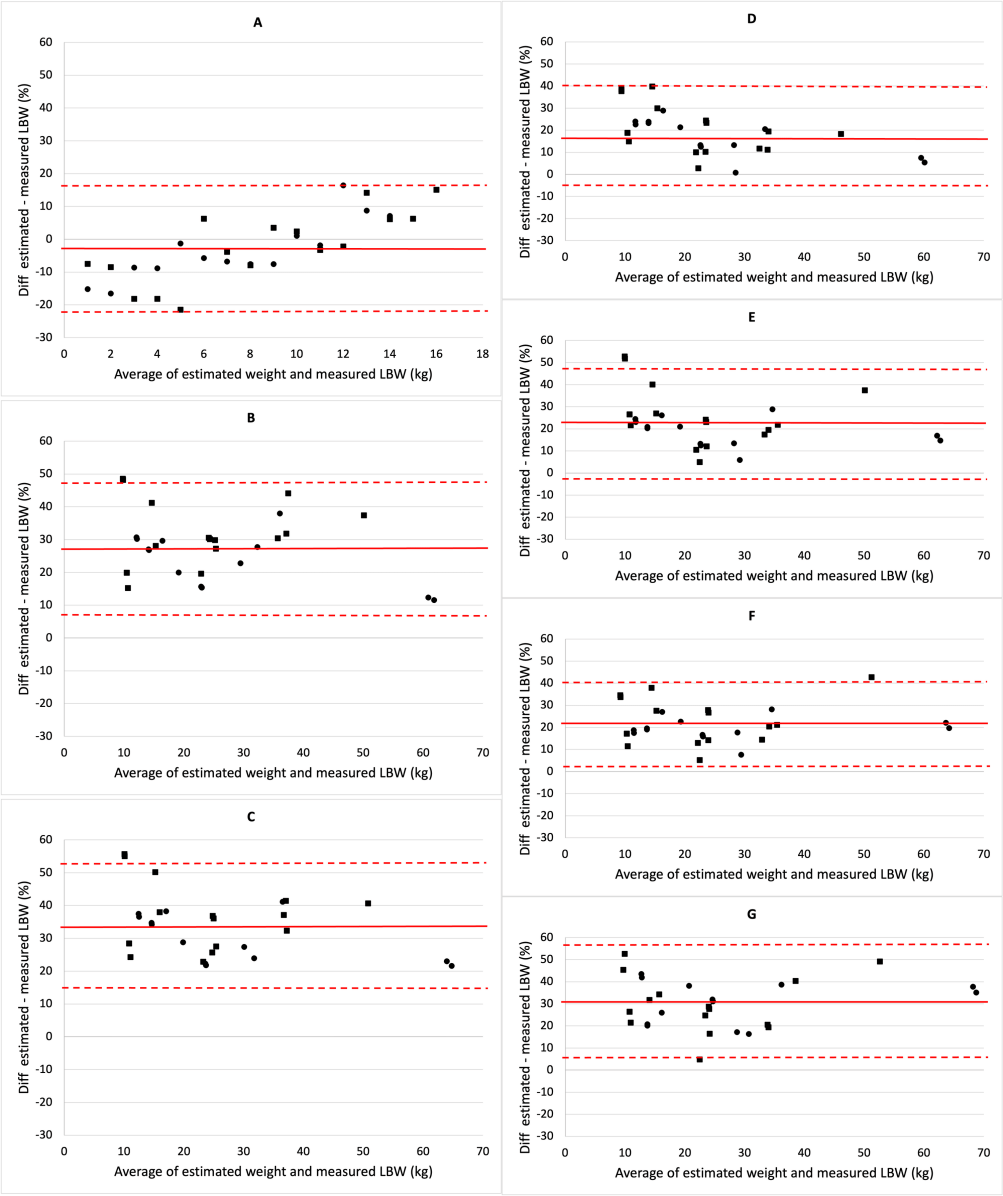
Bland-Altman chart of the LBW and IBW estimation methods (A) Foster formula. (B) Peters formula. (C) Callaghan formula. (D) BMI₅₀ method. (E) McLaren method. (F) Traub-Johnson formula. (G) Moore method. The solid red line represents the MPE, and the dashed red lines represent the 95% limits of agreement. Males are indicated by circular markers, and females by square markers. IBW, ideal body weight; LBW, lean body weight; MPE, mean percentage error

The results of the Bland-Altman percentage error-based analyses are shown in Figure 2. These show the bias and precision of each of the associations between the estimation methods and the reference standard of measured LBW. The substantial bias and the wide limits of agreement are apparent for all methods except for the Foster formula.

**Figure 3:**
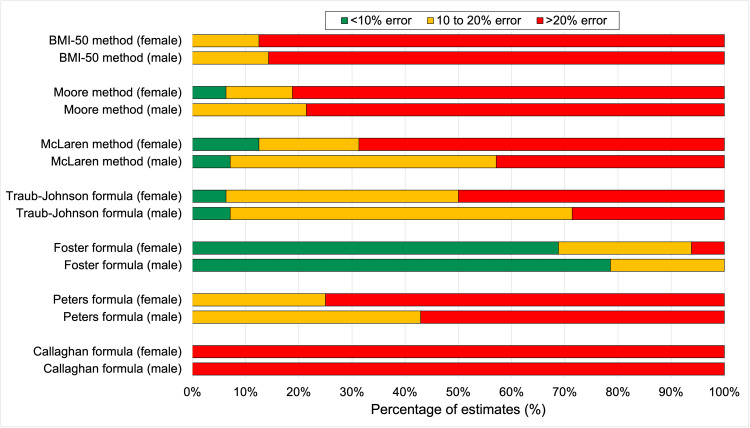
Accuracy of LBW predictions for each of the estimation methods Data for females and males are shown. The green bars indicate the proportion of estimates within 10% of measured LBW. The orange bars represent the proportion of estimates with an error of between 10% and 20%. The red bars indicate critical errors of more than 20%. LBW, lean body weight

Figure 3 summarizes the overall accuracy of the estimating formulas. The proportion of accurate estimates (<10% error), moderately accurate estimates (10-20% error), and critical errors (>20%) is shown. The high rates of critical estimation errors (the red bars) are notable for all the methods with the exception of the Foster formula. Similarly, only the Foster formula showed a satisfactory rate of accurate estimates (the green bars). For most methods, the performance was better in males than in females.

Given the reasonably consistent overestimation of LBW by the IBW formulas, a modifying factor was determined for each method to explore the potential of using these formulas to estimate LBW more accurately. Analyses repeated after applying the modifying factors showed substantially improved estimations of LBW (Table 4). It was not possible to identify a simple correction factor for the Peters and Callaghan formulas.

**Table 4:** Results of the comparisons between the modified IBW formulas and measured LBW A positive value of MPE indicates an overestimation of LBW. The adjusted values were obtained by applying the constant shown in the table to the formula. The results of Fisher’s exact tests between the P10s of the male and female participants are indicated by superscripts: ^o^ OR 3.0 (0.3, 32.8) p = 0.351, ^p^ NaN p = 0.045, ^q^ OR 2.0 (0.3, 13.1) p = 0.464, ^r ^OR 3.0 (0.3, 32.8) p = 0.351. The results of the t-tests between the RMSPE of the male and female participants are indicated by superscripts: ^s^ t-test (two-tailed, independent samples) p = 0.221, ^t^ t-test (two-tailed, independent samples) p = 0.060, ^u^ t-test (two-tailed, independent samples) p = 0.181, ^v^ t-test (two-tailed, independent samples) p = 0.138. IBW, ideal body weight; LBW, lean body weight; LLOA, lower 95% limit of agreement; MPE, mean percentage error; NaN, not a number (cell size too small for analysis); PW10, percentage of estimates within 10% of measured fat-free mass; PW20, percentage of estimates within 20% of measured fat-free mass; ULOA, upper 95% limit of agreement

Parameter	Modified BMI₅₀method			Modified McLaren method			Modified Moore method			Modified Traub-Johnson formula		
	All	Male	Female	All	Male	Female	All	Male	Female	All	Male	Female
Constant	0.85			0.85			0.75			0.8		
MPE	0.9	-1	2.6	3.9	0.4	7.1	-2.5	-2	-2.9	-3.2	-4.7	-1.8
LLOA	-15.7	-14.8	-15.9	-15	-10.4	-15.5	-18.6	-15.5	-21.5	-16.6	-12.6	-18.5
ULOA	17.5	12.8	21	22.9	11.1	29.7	13.7	11.6	15.6	10.3	3.2	14.9
PW10	83.3	92.9^o^	75.0^o^	83.3	100^p^	68.8^p^	80	85.7^q^	75.0^q^	86.7	92.9^r^	81.3^r^
PW20	100	100	100	93.3	100	87.5	96.7	100	93.8	100	100	100
RMSPE	8.4	6.8^s^	9.5^s^	10.3	5.3^t^	13.2^t^	8.5	6.9^u^	9.6^u^	7.4	6.1^v^	8.4^v^

## Discussion

There are significant gaps in our understanding of how different drug dosing scalars relate to underlying body composition in obese children and adolescents. This is critically important to be able to effectively personalize weight-based drug doses for these children, which is, in turn, critically important to ensure safe and effective drug therapy. Our study explored this relationship in a small group of children and adolescents for whom anthropometric data and gold standard measurements of LBW were available.

The concept of ideal IBW was originally developed to assess the nutritional status of children. This was accomplished by comparing a child’s weight to that of an “ideal child” of the same age and height [11]. It was not originally intended for use as a drug dosing scalar. Despite lacking a clearly defined physiological basis and without validation as a dosing scalar, IBW has nevertheless been widely incorporated into pediatric clinical practice for this purpose. IBW is often used with the intention of being a surrogate for LBW, while, in some equations, it serves as a variable to estimate LBW. However, the actual relationship between IBW and LBW has also never been comprehensively established [10]. This knowledge gap is clinically significant, as the correct use of accurate weight-based dose scalars is essential for safe and effective medication dosing in obese children. The current study was designed to address this uncertainty by examining the relationship between various methods of estimating IBW and LBW against measured LBW.

Summary of key findings

This study demonstrated that all the evaluated methods of calculating IBW were substantially different from measured LBW, with mean overestimations ranging from 18% to 30%. None of the IBW methods achieved an acceptable predictive accuracy. Amongst the LBW estimation methods, the Foster formula performed with reasonable accuracy, while the Peters and Callaghan methods performed very poorly. These findings align partly with prior work by Ross et al., who compared BMI₅₀ IBW values with the Peters and Foster methods and found apparent agreement between the three, although no reference standard for LBW was included [9]. A closer inspection of their data suggested considerable variation, with notably better agreement between the Foster and Peters methods than between either and the BMI₅₀ approach.

Interpretation and comparison with previous studies

The current findings confirm that the Foster method is accurate but is more computationally complex than other methods, making it less practical for routine or emergency use without computerized support. The Peters method, by contrast, was derived from a limited dataset that included few obese children, likely explaining its poorer performance and larger estimation errors than initially reported [20]. The Callaghan formula proved inaccurate, perhaps due to its underlying assumption of the relationship between LBW and IBW in obese children. This assumption was not compatible with the current data [10]. Our results, therefore, challenge the premise that IBW formulas can serve as reliable surrogates for LBW in obese children. None of the existing IBW equations accurately predicted LBW, and if IBW is to be used for this purpose, substantial modification of the formulas would be required, or the “best” method would need to be identified and studied further, as estimations may differ significantly between different methods [12,21]. IBW formulas have been compared with each other, but generally not against any physiologically meaningful standard [12,18,22,23]. Appelbaum and Clarke recommended that the BMI₅₀ method be adopted as the preferred method because of its relative simplicity and consistency [18]. Interestingly, we found that all the IBW methods could be adapted with a simple adjustment factor to predict LBW with a high degree of accuracy. However, if IBW is used to function as a surrogate for LBW, its biological validity must further be established, specifically whether IBW maintains a consistent relationship with body composition across age, sex, and body habitus [11]. However, the LBW formulas of Peters and Callaghan could not be improved in the same way as the IBW formulas.

Clinical and practical implications

The theoretical mismatch between IBW (a construct originally developed for nutritional assessment) and LBW (which reflects metabolic and pharmacokinetic relevance) strengthens the conceptual framing of why IBW could be expected to perform poorly in predicting LBW. It also highlights the folly in repurposing an estimation method without establishing its validity for the repurposed task. The findings in this study suggest that IBW should not be repurposed for drug dosing calculations. Thus, methods of LBW need to be adopted in greater measure in clinical and research settings to ensure optimum drug delivery.

The findings also have practical implications for pediatric drug dosing. Although this was a relatively small study, the data suggest that the Foster formula can be used reliably to estimate LBW in obese children, whereas the Peters and Callaghan methods should not be recommended. The BMI₅₀ approach to determine IBW, while not highly accurate, was the least inaccurate of the IBW-based methods and remains the most reproducible for point-of-care applications. It is particularly suited to be adapted for use in length-based emergency tools such as the PAWPER tape and the Broselow tape [6], which depend on consistent, scalable estimation methods.

Future directions

The results highlight an important need for further research to refine and clarify the role of IBW and LBW in pediatric dosing, particularly in obese children. A valid dose-scaling method must be simple, reproducible, and equally accurate across sexes and a broad range of ages and body types. Moreover, if IBW is to retain its role in clinical practice, its physiological and pharmacokinetic foundations must be rigorously validated in obese children. Future work should therefore focus on developing modified or hybrid models that maintain the simplicity of IBW estimation while achieving the physiological relevance and predictive precision of LBW-based methods. For emergency care settings, methods that do not require calculations or complex measurements should be developed.

Limitations

This study has several important limitations. First, the most significant limitation is the small sample size (n = 30) drawn from a single center, which restricts the precision of estimates and limits generalizability, particularly across age ranges, degrees of obesity, and diverse ethnic or physiological populations. The small sample size, therefore, affects the confidence in the findings of the performance differences between formulas. These will need to be verified in larger, more diverse studies. Second, several of the predictive equations evaluated were originally developed using samples with different age mixes, which may have biased their performance in our pediatric sample. Third, as this was a retrospective secondary analysis, we were limited to the available data and could not control for all potential confounders, such as disease-related changes in body composition. This could introduce the possibility of selection bias, which could result in the potential underrepresentation of older adolescents or those with severe obesity. Finally, although the study identified correction factors that improved IBW-based LBW predictions, these adjustments were derived empirically from a small dataset and require validation in larger, prospective, and more diverse cohorts before they can be recommended for clinical use.

## Conclusions

We found substantial discrepancies between existing methods of estimating IBW and LBW and measured LBW in obese children and adolescents. None of the IBW formulas accurately predicted measured LBW, suggesting that IBW should be reconsidered as a surrogate for LBW without modification or validation. Of the evaluated LBW formulas, only the Foster equation demonstrated acceptable accuracy, though its complexity may limit clinical practicality without computational assistance. The Peters and Callaghan formulas performed poorly, emphasizing the need for more representative derivation datasets. Given the small sample size in this study, the findings cannot be regarded as definitive. However, the findings definitely suggest that further studies are essential for further validation in larger, more diverse cohorts before translating results into clinical standard-of-care guidance. Future work should also focus on refining or developing hybrid models that combine the ease of IBW estimation with the physiological accuracy of LBW prediction, ensuring both safety and feasibility in pediatric drug dosing across diverse body types.
